# Baseline pain medication is associated with longer duration of high adherence in a three-month digital treatment program for hip and knee osteoarthritis

**DOI:** 10.1016/j.ocarto.2025.100727

**Published:** 2025-12-11

**Authors:** Leif E. Dahlberg, Simon P. Rowland, Jack T. Pearson, L. Stefan Lohmander, Ali Kiadaliri

**Affiliations:** a Department of Clinical Sciences Lund, Orthopaedics, Lund University, Lund, Sweden; b Haleon UK Trading Limited, Building 5, 1 st Floor, The Heights, Weybridge, Surrey, KT13, UK; c Haleon UK Trading Limited, Building 5, 1 st Floor, The Heights, Weybridge, Surrey, KT13 0NY, UK

**Keywords:** eHealth, Physiotherapy, Osteoarthritis, Pain medication

## Abstract

**Objective:**

To investigate whether baseline use of pain medication is associated with program adherence during a three-month digital treatment program for individuals with hip or knee osteoarthritis (OA).

**Design:**

An observational cohort study using registry data on weekly participant adherence from 33078 participants enrolled in a digital education and exercise therapy program. Poor adherence was defined as completing less than 80 % of the approximately 20 prescribed weekly activities for two consecutive weeks during the 13-week treatment period. Baseline analgesic use was categorized into six groups: no medication, paracetamol (with/without dietary supplements), NSAIDs (with/without supplements), paracetamol combined with NSAIDs (with/without dietary supplements), dietary supplements only, and opioids (with/without other medications). Interval-censored parametric survival models adjusted for baseline characteristics were used for statistical analysis. In sensitivity analyses, alternative definitions of poor adherence were used. A complete case analysis was conducted as a subgroup analysis.

**Results:**

Compared to the no-medication group, individuals taking paracetamol—either alone (hazard ratio 0.94, 95 % CI 0.91, 0.98) or in combination with NSAIDs (0.91, 0.87, 0.94) reached poor adherence later. In contrast, opioid users (hazard ratio 1.12, 95 % CI 1.06, 1.19) reached poor adherence earlier. Adjusted median days to reach poor adherence ranged from 39.2 (95 % CI 36.9, 41.4) for opioids to 49.2 (47.7, 50.7) for paracetamol + NSAIDs users. Alternative definitions of poor adherence and a complete-case analysis generally yielded similar findings.

**Conclusion:**

Baseline use of paracetamol, alone or with NSAIDs, was associated with longer time to reach poor adherence, whereas opioid use predicted poor adherence earlier.

## 1 Introduction

Physical activity and pharmacological treatment are both well-established, evidence-based interventions for managing osteoarthritis (OA)-related symptoms. International guidelines consistently recommend exercise and education as first-line OA management with the addition of pharmacological support as needed [1,2]. First-line interventions can be delivered either in person or digitally. Clinical studies have shown that digital therapy providing evidence-based OA treatment via an app are associated with clinically meaningful reductions in pain, improved physical function, improved symptom state, and in some cases a reduced need for joint replacement surgery [[3], [4], [5]].

Digital therapy, also referred to as telehealth or e-physiotherapy, involves delivering assessment, education, and treatment remotely through secure digital communication. It enables direct interaction between patient and licensed healthcare professional in real time or asynchronously via video, chat, or mobile applications. This approach allows evidence-based physiotherapy to be provided independent of location, offering flexible access, continuous monitoring, and personalized support comparable to traditional in-person care.

Joint Academy is a digital platform providing first-line evidence-based physiotherapy for patients with musculoskeletal pain. Regarding treatment for mild to moderate OA of the hip and knee, patients access the program via smartphone or tablet and are connected to a licensed physiotherapist through video and chat. Treatment includes individualized daily video exercises, educational lessons, progress tracking, and reminders. Patients typically perform two to four short exercises per day following a “little and often” principle, with gradual progression in intensity. The physiotherapist continuously adapts the program to each patient's needs throughout treatment.

Adherence to digital OA treatments differs between delivery modes and tends to decrease over time [[6], [7], [8]]. Despite analysis of a wide range of demographic and clinical predictors, only a small proportion of the variation in adherence can be explained, suggesting influence of behavioural or unmeasured patient-specific variables.

Commonly used pharmacological treatments for OA include over-the-counter paracetamol and non-steroidal anti-inflammatory drugs (NSAIDs), administered either orally or topically [1,2]. Although NSAIDs are recommended in most clinical guidelines for symptom relief, paracetamol has limited and inconsistent support due to small effect sizes, and opioids are discouraged because of poor tolerability and risk of adverse effects [1,2]. Dietary supplements such as glucosamine or rosehip extract are also frequently used, despite a lack of convincing evidence for clinical benefit [1,2].

Pharmacological treatments may facilitate participation in exercise-based rehabilitation by reducing pain and stiffness, thus lowering the perceived barriers to movement and self-management. Improved symptom control could enhance motivation and self-efficacy—factors known to support adherence to physical activity programs [9]. However, little is known about whether baseline analgesic use contributes to maintaining engagement in digital rehabilitation over time, or whether certain medications may hinder participation due to side effects or unrealistic expectations of pain relief.

This study investigated whether baseline use of pain medication—including paracetamol, NSAIDs, opioids, and dietary supplements—was associated with adherence over a three-month digital treatment program for individuals with hip or knee OA. We hypothesized that baseline use of pain medication would be associated with longer time to reach poor adherence in a digital program delivering exercises and educational lessons for OA.

## 2 Methods

### 2.1 Study design

This was an observational cohort study according to STROBE, using registry data from participants with knee or hip OA enrolled in a digital education and exercise therapy, known as Joint Academy® [5]. Participants are geographically distributed across Sweden and access the program remotely from their homes. The digital platform provides first-line, evidence-based physiotherapy for patients with musculoskeletal pain. Patients access the program via smartphone or tablet and are connected to a licensed physiotherapist through video and chat. Treatment includes individualized daily video exercises, educational lessons, and progress tracking. To register and begin treatment, patients downloaded the app and securely logged in to access activities including exercises, lessons, and questionnaires. Exercises and lessons had to be checked off upon completion to proceed, thereby confirming adherence to the prescribed treatment and enabling progress monitoring. Data are self-reported through the mobile application, collected at baseline, during treatment, and at the 3-month follow-up.

### 2.2 Participants

Eligible participants were adults aged ≥18 years with clinically confirmed hip or knee OA who enrolled in the digital program between January 2022 and December 2024 (n = 34935). Of these, 1804 left the program prior to week 1 and 53 did not answer the health questionnaire (the main exposure of interest) leaving 33,078 participating in the study. Diagnoses were confirmed through referral documentation or structured phone interviews by licensed physiotherapists, based on NICE guidelines [10] and Swedish national criteria [11]. Participants were recruited through healthcare providers (e.g., physiotherapists, orthopaedic surgeons) or self-enrolled via online advertisements and social media. We excluded 53 persons with missing data on analgesics.

### 2.3 Outcome

#### 2.3.1 Poor adherence

Adherence was calculated as the percentage (0–100) of completed prescribed activities, including exercises, lessons, and quizzes. This was measured weekly during the 13-week participation period. Participants performed two assigned exercises per day, and received educational lessons every second to third day, resulting in approximately 20 prescribed activities per week.

In our main analysis, we defined *poor adherence* as two consecutive weeks with adherence to less than 80 % of the prescribed activities during treatment. This threshold was chosen following the Swedish clinical practice guideline recommendations for first-line treatments of knee and hip OA [10]. The rationale for using a two-week window was that adherence often fluctuates over time, a participant with an average adherence of 80 % may alternate between periods of complete inactivity and consistent engagement. Measuring time to poor adherence captures these dynamic patterns more accurately than a static adherence rate.

In sensitivity analyses, we used three alternative poor adherence levels during one week in treatment: <80 %, <50 %, or 0 % adherence. If a person had a week with missing adherence level (e.g. dropping out from the program), then adherence level was considered as 0 % for that week. Alternative definitions of poor adherence were analysed separately.

#### 2.3.2 Analgesic use

At first week, participants reported whether they have used any medication for joint pain in the past month. Medication classes included paracetamol, NSAIDs, opioids, other prescribed medications and supplements. Examples included in the questionnaire were: paracetamol; NSAIDs (ibuprofen, naproxen, diclofenac, celecoxib); opioids with examples provided of common Swedish brand names to aid the participant; and dietary supplements (rosehip extract, glucosamine, turmeric).

Based on the distribution of responses and strength of the analgesic, we categorized analgesic use into six groups: no medication, paracetamol with or without dietary supplements, NSAIDs with or without dietary supplements, paracetamol and NSAIDs with or without dietary supplements, only dietary supplements, and opioids with or without other medications.

#### 2.3.3 Covariates (please see supplement for references)

All models were adjusted for the following baseline variables: age, sex, education, numerical rating scale pain (0 = no pain, 10 = worst pain), body mass index, sleep rates (0–10, very poorly–very well), EQ-5D-5L index score, readiness to do exercise (0–10, not at all ready–extremely ready), activity impairment (0–10, OA had no effect on my daily activities–OA completely prevented me from doing my daily activities), comorbidity (diabetes, lung disease, balance issues, rheumatoid arthritis, cardiovascular disease), physical activity level according to the Swedish National Board of Health and Welfare, KOOS-12/HOOS-12 total score, walking difficulty (yes/no), fear of movement (yes/no), and willingness to undergo surgery (yes/no/don't know).

#### 2.3.4 Statistical analysis

Baseline characteristics were summarized using means and standard deviations for continuous variables, and frequencies and percentages for categorical variables.

To explore the associations between baseline analgesic group and time to reach poor adherence, we used parametric survival analyses with interval-censoring. Our data is interval-censored since adherence was measured at the end of the week. Individuals reaching poor adherence were considered as interval-censored while those who didn't reach poor adherence were considered as right censored.

We estimated different distributions and based on the Bayesian information criterion selected Weibull distribution as our preferred distribution. The results are presented as hazard ratios (HR) with 95 % confidence intervals. In our case, an HR > 1 suggests a shorter time from baseline to reach poor adherence compared with the no medication reference group, while an HR < 1 suggests the opposite. We conducted a subgroup analysis among individuals who participated throughout the 13-week program (complete case analysis). The study was approved by the Swedish ethical review authority.

## 3 Results

33,078 patients with a mean (SD) age of 64.6 (10.1) years, and 72.4 % females were included (Table 1). Among these, 35.3 % were not using any medication, 57.5 % were using NSAIDs, paracetamol or a combination of them (with or without supplements), 1.9 % were using supplements solely, and 5.2 % were using opioids with or without other medications. The distribution of analgesic use at baseline is presented in Table S1 in the supplement.

**Table 1:** Baseline characteristics.

	No medication	Paracetamol	NSAIDs	Opioids	Paracetamol + NSAIDs	Supplements	Total
N (%)	11689 (35.3)	7215 (21.8)	4636 (14.0)	1733 (5.2)	7179 (21.7)	626 (1.9)	33078 (100)
Age, mean (SD)	64.6 (10.5)	67.8 (9.7)	62.1 (9.2)	63.7 (10.4)	63.2 (9.6)	63.3 (9.6)	64.6 (10.1)
Female, n (%)	7729 (66.1)	5525 (76.6)	3244 (70.0)	1275 (73.6)	5771 (80.4)	412 (65.8)	23957 (72.4)
Education, n (%)							
Pre-secondary	903 (7.7)	959 (13.3)	276 (6.0)	207 (11.9)	504 (7.0)	70 (11.2)	2920 (8.8)
Upper-secondary up to high school	2835 (24.3)	2108 (29.2)	1209 (26.1)	531 (30.6)	1960 (27.3)	196 (31.3)	8839 (26.7)
Post-secondary up to bachelor's degree	5793 (49.6)	3271 (45.3)	2336 (50.4)	797 (46.0)	3599 (50.1)	280 (44.7)	16076 (48.6)
Postgraduate	2158 (18.5)	877 (12.2)	815 (17.6)	198 (11.4)	1116 (15.6)	80 (12.8)	5244 (15.9)
Sleep rate (0–10), mean (SD)	6.6 (2.2)	6.0 (2.2)	6.3 (2.2)	5.1 (2.4)	5.8 (2.2)	6.2 (2.3)	6.2 (2.2)
Motivation (0–10), mean (SD)	9.2 (1.4)	9.2 (1.4)	9.4 (1.2)	9.1 (1.5)	9.3 (1.3)	9.1 (1.5)	9.3 (1.4)
NRS pain (0–10), mean (SD)	4.9 (1.8)	6.1 (1.6)	5.8 (1.7)	6.9 (1.6)	6.2 (1.6)	5.8 (1.9)	5.7 (1.8)
BMI, mean (SD)	26.5 (4.5)	27.7 (5.0)	27.3 (4.9)	29.5 (6.2)	28.1 (5.3)	27.7 (5.0)	27.4 (5.0)
Comorbidity (0–5), mean (SD)	0.3 (0.6)	0.5 (0.7)	0.2 (0.5)	0.6 (0.8)	0.3 (0.6)	0.3 (0.6)	0.3 (0.6)
KOOS-12/HOOS-12 total score (0–100), mean (SD)	58.9 (14.8)	48.1 (14.3)	51.6 (14.4)	37.6 (14.8)	45.8 (14.5)	50.2 (16.2)	51.4 (15.9)
Swedish EQ-5D-5L index (−0.31–1), mean (SD)	0.88 (0.14)	0.80 (0.19)	0.83 (0.17)	0.63 (0.27)	0.77 (0.21)	0.80 (0.21)	0.82 (0.19)
Recommended level of physical activity, n (%)	6437 (55.1)	3125 (43.3)	2544 (54.9)	623 (36.0)	3390 (47.2)	304 (48.6)	16423 (49.7)
Walking difficulty, n (%)	6483 (55.5)	5611 (77.8)	3328 (71.8)	1545 (89.2)	5797 (80.8)	459 (73.3)	23223 (70.2)
Fear of movement, n (%)	2728 (23.3)	1611 (22.3)	1396 (30.1)	490 (28.3)	1898 (26.4)	166 (26.5)	8289 (25.1)
Wish for surgery, n (%)							
No	8405 (71.9)	3791 (52.5)	2731 (58.9)	613 (35.4)	3567 (49.7)	332 (53.0)	19439 (58.8)
Yes	1605 (13.7)	1931 (26.8)	1031 (22.2)	718 (41.4)	2029 (28.3)	157 (25.1)	7471 (22.6)
Don't know	1679 (14.4)	1493 (20.7)	874 (18.9)	402 (23.2)	1583 (22.0)	137 (21.9)	6168 (18.7)
Activity impairment (0–10), mean (SD)	3.7 (2.3)	5.0 (2.2)	4.6 (2.3)	6.1 (2.0)	5.2 (2.2)	4.5 (2.4)	4.5 (2.4)

At baseline, the “no medication” group generally had better patient-reported outcomes compared with medication users, with lower BMI, fewer walking difficulties, and less wish for surgery. On the other hand, individuals taking opioids had poorer patient-reported outcomes, higher BMI, lower physical activity levels, and greater willingness to undergo surgery compared to other medication groups (Table 1). Among participants, 85 individuals had missing values on covariates (no medication: 41; paracetamol: 14; NSAIDs:10; opioids:3; paracetamol + NSAIDs: 17) and were excluded from further analysis.

During follow-up, 24,740 (75.0 %) individuals reached poor adherence defined as two consecutive weeks with adherence <80 %, including those who withdrew. Across medication groups, this ranged from 73.1 % in the paracetamol group to 82.2 % in the opioid group (Table 2).

**Table 2:** Number (%) of individuals reaching alternative definitions of poor adherence, stratified by medication category.

	2 consecutive weeks with adherence<80 %	1 week with adherence<80 %	1 week with adherence<50 %	0 % adherence
No medication	8763 (75.2)	10726 (92.1)	9579 (82.2)	8078 (69.4)
Paracetamol	5263 (73.1)	6512 (90.4)	5838 (81.1)	4982 (69.2)
NSAIDs	3528 (76.3)	4267 (92.2)	3811 (82.4)	3186 (68.9)
Opioids	1422 (82.2)	1629 (94.2)	1501 (86.8)	1294 (74.8)
Paracetamol + NSAIDs	5297 (74.0)	6498 (90.7)	5808 (81.1)	4938 (69.0)
Supplements	467 (74.6)	569 (90.9)	497 (79.4)	419 (66.9)
Total	24740 (75.0)	30201 (91.5)	27034 (81.9)	22897 (69.4)

**Fig. 1:**
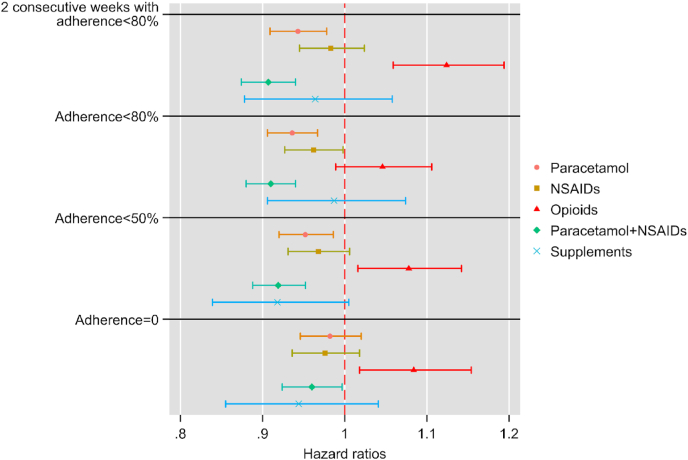
Adjusted hazard ratios (95 % confidence intervals) for the associations between baseline medication use and time to reach poor adherence in the digital osteoarthritis treatment program. The main analysis (top panel) defines poor adherence as two consecutive weeks with adherence <80 %. Sensitivity analyses (lower panels) applied alternative definitions of poor adherence based on one week of adherence <80 %, <50 %, or 0 %. Participants with missing adherence data (e.g., dropout) were considered to have 0 % adherence for that week. Hazard ratios (HR) > 1 indicate a shorter time to reach poor adherence, whereas HR < 1 indicate a longer time to remain adherent.

The estimated HRs suggested that compared with the no-medication group, paracetamol and paracetamol + NSAIDs groups reached poor adherence later, while those taking opioids reached poor adherence earlier (Fig. 1). The associations for NSAIDs-only and supplements groups were inconclusive. The hazard ratio observed for the paracetamol + NSAID group corresponds to five more days of adherence compared to the non-medication group and ten more days compared to the opioid group (Tables S2 and S3 Supplement), which may represent up to 25-50 additional completed activities.

Similar patterns were generally observed for alternative definitions of poor adherence, with the main exception when 0 % adherence was defined as poor adherence, where paracetamol was no longer conclusively associated with delay in reaching poor adherence. Subgroup analysis among those participating throughout the 13-week program (n = 12,640) was generally consistent with our main analysis, with the main exception being the lack of a conclusive association between opioid use and time to poor adherence (Fig. S1 in the supplement).

## 4 Discussion

In the present study, we tested the hypothesis that baseline use of pain medication would be associated with longer time to reach poor adherence in a digital first-line exercise and education program delivering exercises and educational lessons for OA. Individuals taking paracetamol, either alone or in combination with NSAIDs at enrolment, maintained good adherence for a longer period compared with those not taking any medication. In contrast, opioid users reached poor adherence earlier.

During follow-up, 75 % of the participants reached poor adherence, defined as two consecutive weeks with <80 % completion, including those who withdrew. While this proportion appears high, it reflects real-world engagement rather than treatment failure. In digital self-managed programs, temporary drops in participation are expected and differ from adherence patterns in traditional, physiotherapist-supervised programs where attendance is recorded at in-person visits. Adherence often fluctuates over time with periods of nonadherence alternating with consistent engagement. Evaluating time spent within the desired adherence range therefore provides a more nuanced understanding of patient behaviour in digital rehabilitation.

Our definition of adherence, based on the percentage of completed digital activities (exercises, lessons, and quizzes) for two weeks, was selected to be objective, automatically measurable, and applicable in large-scale registry data. This contrasts with therapist-reported attendance in supervised settings [7]. The digital format enables continuous monitoring of behaviour. Adherence remains a multidimensional construct without universal consensus [6]; our operational definition of poor adherence (<80 % completion for two consecutive weeks) aligns with Swedish first-line OA care guidelines and provides a reproducible, clinically meaningful measure suited for real-world digital rehabilitation [11].

The relationship between adherence and clinical outcomes is complex. While Marriott et al. [12] found no overall association between adherence and improvements in pain or physical function in a meta-analysis, several individual studies have demonstrated that higher adherence predicts better outcomes. Pisters et al. showed that greater adherence to home-based exercise significantly improved pain, function, and performance over 60 months, and Bennell et al. reported similar findings for an internet-delivered exercise and pain-coping program in chronic knee pain ([9,13]. These observations emphasize that not only the content but also the consistency of participation is key for achieving optimal results in OA management. The observed hazard ratio for the paracetamol + NSAID group corresponds to approximately five additional days of adherence, or about 25 extra completed activities during the 13-week program. Although modest, this effect may be clinically meaningful given the established link between adherence and improved outcomes in OA.

The finding that paracetamol and the combination of paracetamol and NSAID is associated with better adherence in people with OA symptoms in the digital treatment program can have several explanations. By reducing pain and stiffness, evidence-based pain medications according to guidelines may facilitate exercise and daily activities. The experience of improved symptom relief and more tolerable symptoms may lower perceived barriers to participation. It could enhance motivation and self-efficacy, enabling individuals to feel more capable of completing prescribed activities. In addition, better short-term pain control may provide immediate positive feedback, reinforcing continued adherence to the program over time. Participants without pain medication had better patient-reported outcomes at baseline. This supports the importance of appropriate pain management to enable individuals with higher symptom burden to engage fully in digital rehabilitation.

Individuals using opioids at baseline reached poor adherence earlier, and this may reflect factors beyond those adjusted for in the present study. Opioid-related side effects such as fatigue, sedation, or cognitive impairment could hinder participation in exercise-based digital programs [14,15]. Opioid use may also indicate lower self-efficacy or a more passive coping style, both of which are less compatible with self-managed digital rehabilitation [16]. Moreover, opioids have shown poor tolerability, frequent adverse events, and limited clinical benefit in osteoarthritis pain management, particularly compared with non-opioid analgesics [[17], [18], [19]]. Together, these findings support the notion that individuals using opioids are less likely to engage successfully in long-term, self-directed exercise interventions.

### 4.1 Limitations

The study population consists of individuals who voluntarily enrolled in a digital treatment program, which may select for patients who are more motivated, technologically literate, or have better access to digital tools compared to the broader OA population. The adherence patterns observed here may not fully reflect those seen in traditional in-person care. In addition, socio-economic factors, regional access to healthcare, and cultural attitudes toward digital health interventions could influence participation and outcomes further limiting the external validity.

Another limitation is that all data, including medication use, pain levels, and adherence measures, were self-reported, which may introduce recall bias or reporting bias. The observational design of this study also restricts the ability to draw causal inferences. Unmeasured confounding variables, such as individual behavioural factors or psychosocial influences, may have affected both analgesic use and adherence outcomes.

In conclusion, baseline use of paracetamol, alone or with NSAIDs, was associated with longer time to reach poor adherence, whereas opioid use predicted poor adherence earlier. Given the high and increasing OA disease burden, even a modest improvement in adherence could translate into meaningful population-level health benefits. Our findings need confirmation in studies specifically designed to examine the role of oral and topical pain medication, adherence patterns, and other relevant outcomes and demographic factors.

## Credit authorship contribution statement

All authors contributed to the conception and design of the study, analysis and interpretation of data, critical revision of the manuscript for important intellectual content, and final approval of the version to be submitted.

LD: Drafting of the article.

AK: Statistical expertise.

SR and JT: Obtaining funding.

LD takes responsibility for the integrity of the work as a whole, from inception to finished article.

## Declaration of Generative AI and AI-assisted technologies in the writing process

During the preparation of this work the author used ChatGPT in order to improve the English language. After using this tool, the authors reviewed and edited the content as needed and take full responsibility for the content of the publication.

## Funding

This research received a grant from Haleon.

## Declaration of competing interest

LD reports receiving a research grant for this study from Haleon, serving on Haleon's Advisory Board for the Digital RWE Working Group, holding stock and stock options in Arthro Therapeutics, and serving as Chief Medical Officer at Joint Academy.

AK reports being a part-time (7.5 %) scientific advisor for Arthro Therapeutics.

SL reports consulting fees from: Arthro Therapeutics AB and Synartro AB, Sweden, and Arthrex, chair of Data and Safety Monitoring Board, and member of DSMB, Arthritis Foundation, USA.

SR and JP report stock and stock options in Haleon UK Trading Ltd and stock in Natural Cycles Nordic AB. Both are employees of Haleon UK Trading Ltd.

The Joint Academy team was responsible for design, conduct and oversight of the research study. The scope of the collaboration with Haleon was formally agreed and defined through a legal contract between the two parties. In addition to provision of funding, this agreement allowed the Haleon scientific team to review and comment on study documents including the study proposal, study report and final drafts of the manuscript. The data collection and data analysis were conducted fully independently by the Joint Academy research team. In all research related matters the Joint Academy research team held the final decision for approval and manuscript submission.
