# Supplementary material for: Baseline pain medication is associated with longer duration of high adherence in a three-month digital treatment program for hip and knee osteoarthritis
Source: Osteoarthr Cartil Open. 2025 Dec 11;8(1):100727. doi: 10.1016/j.ocarto.2025.100727 (PMC12795999; doi:10.1016/j.ocarto.2025.100727)
Supplement: Multimedia component 1 [file mmc1.docx]

**Baseline Variables and References**

Models were adjusted for the following baseline variables:

- Age and sex

- Education, self-reported: What is the highest level of education that you have completed? (Pre-secondary education 9 years, Upper secondary education maximum 2 years, High school education 3 years or more, Post-secondary education shorter than 3 years, Bachelor’s degree or equivalent, Master’s degree or equivalent, Doctoral degree or equivalent

- Pain measured using an 11-point Numeric Rating Scale (0 = no pain, 10 = worst possible pain) [1]

- Activity impairment (“During the past 7 days, how much did knee/hip OA affect your ability to do your regular daily activities?”, 0 = no effect, 10 = completely preventing daily activities) [2]

- Body mass index (BMI)

- Sleep quality: How do you usually sleep? (0 = very poorly, 10 = very well)

- EQ-5D-5L index score (using the Swedish value set) [3]

- Readiness to exercise: How ready are you to start doing exercises on a daily basis? (0 = not at all ready, 10 = extremely ready)

- Self-reported doctor-diagnosed comorbidities (diabetes, lung disease, balance issues, rheumatoid arthritis, cardiovascular disease)

- Physical activity Two validated categorical questions from the Swedish National Board of Health and Welfare (NBHW) are used to measure the level of physical activity in the digital program. One question is about everyday physical activity and another one about exercise.
Everyday physical activity (moderate physical activity) is measured using the following question: “How much time do you spend in a typical week on daily physical activity that is not exercise, such as walking, cycling or gardening?”. There are 7 possible options coded 1 to 7: 1) 0 min, 2) less than 30 min, 3) 30–60 min, 4) 61–90 min, 5) 91–150 min, 6) 151–300 min, and 7) more than 300 min.
The question for measuring exercise (vigorous physical activity) is: “During a regular week, how much time do you spend exercising on a level that makes you short winded, for example running, group fitness classes, or ball sports?”. There are 6 possible options coded 1-6: 1) 0 min, 2) less than 30 min, 3) 30–60 min, 61–90 min, 91–120 min, and more than 120 min. A total physical activity score is computed by multiplying the score for exercise time by two and adding this to the score for everyday physical activity time, resulting in an ordinal scale ranging from 3 to 19. According to the NBHW, a total physical activity score≥11 represents the cut-off score for achieving the recommended level of physical activity (i.e. 150 minutes of moderate-to-vigorous PA per week). We used this cut-off score to classify the participants as reaching the recommended physical activity level (yes/no).[4]

- KOOS-12/HOOS-12 total score [5,6]

- Walking difficulty (yes/no): Do you have difficulties walking due to problems in your hips or knees?

- Fear of movement (yes/no): Are you concerned that your Joint may be damaged by physical activity or exercise?

- Willingness to undergo surgery (“Are your symptoms so severe that you wish to undergo surgery in your knee/hip?” yes/no/don’t know)

**References**

1. Williamson A, Hoggart B. Pain: a review of three commonly used pain rating scales. J Clin Nurs. 2005;14(7):798-804. doi:10.1111/j.1365-2702.2005.01121.x

2. Reilly MC, Zbrozek AS, Dukes EM. The validity and reproducibility of a work productivity and activity impairment instrument. Pharmacoeconomics. 1993;4:353-65.

3. Sun S, Chuang LH, Sahlén KG, Lindholm L, Norström F. Estimating a social value set for EQ-5D-5L in Sweden. Health Qual Life Outcomes. 2022;20:167. doi:10.1186/s12955-022-02083-w

4. Olsson SJ, Ekblom O, Andersson E, Börjesson M, Kallings LV. Categorical answer modes provide superior validity to open answers when asking for level of physical activity: A cross-sectional study. Scand J Public Health. 2016;44:70-6.

5. Gandek B, Roos EM, Franklin PD, Ware JE Jr. A 12-item short form of the Knee injury and Osteoarthritis Outcome Score (KOOS-12): tests of reliability, validity and responsiveness. Osteoarthritis Cartilage. 2019;27:762-70.

6. Gandek B, Roos EM, Franklin PD, Ware JE Jr. A 12-item short form of the Hip disability and Osteoarthritis Outcome Score (HOOS-12): tests of reliability, validity and responsiveness. Osteoarthritis Cartilage. 2019;27:754-61.

**Table S1. Medication use patterns at enrolment**

| Pattern | N | Percent | Analyzed group |
| --- | --- | --- | --- |
| No medication | 11689 | 35.3 | No medication |
| Only paracetamol | 5816 | 17.6 | Paracetamol |
| Only NSAIDs | 3933 | 11.9 | NSAIDs |
| Only opioids | 259 | 0.8 | Opioids |
| Only supplements | 626 | 1.9 | Supplements |
| Paracetamol + supplements | 1399 | 4.2 | Paracetamol |
| NSAIDs + supplements | 703 | 2.1 | NSAIDs |
| Opioids + supplements | 50 | 0.2 | Opioids |
| Paracetamol + NSAIDs | 5731 | 17.3 | Paracetamol + NSAIDs |
| Paracetamol + opioids | 515 | 1.6 | Opioids |
| NSAIDs + opioids | 130 | 0.4 | Opioids |
| Paracetamol + NSAIDs + supplements | 1448 | 4.4 | Paracetamol + NSAIDs |
| Paracetamol + opioids + supplements | 145 | 0.4 | Opioids |
| Paracetamol + NSAIDs +opioids | 441 | 1.3 | Opioids |
| NSAIDs + opioids + supplements | 42 | 0.1 | Opioids |
| Paracetamol + NSAIDs +opioids + supplements | 151 | 0.5 | Opioids |

**Figure S1. Crude hazard ratios (95% confidence intervals) for the associations between baseline medication use and time to reach poor adherence in the digital osteoarthritis treatment program.**The main analysis (top panel) defines poor adherence as two consecutive weeks with adherence <80%. Sensitivity analyses (lower panels) applied alternative definitions of poor adherence based on one week of adherence <80%, <50%, or 0%. Participants with missing adherence data (e.g., dropout) were considered to have 0% adherence for that week. Hazard ratios (HR) >1 indicate a shorter time to reach poor adherence, whereas HR <1 indicate a longer time to remain adherent.


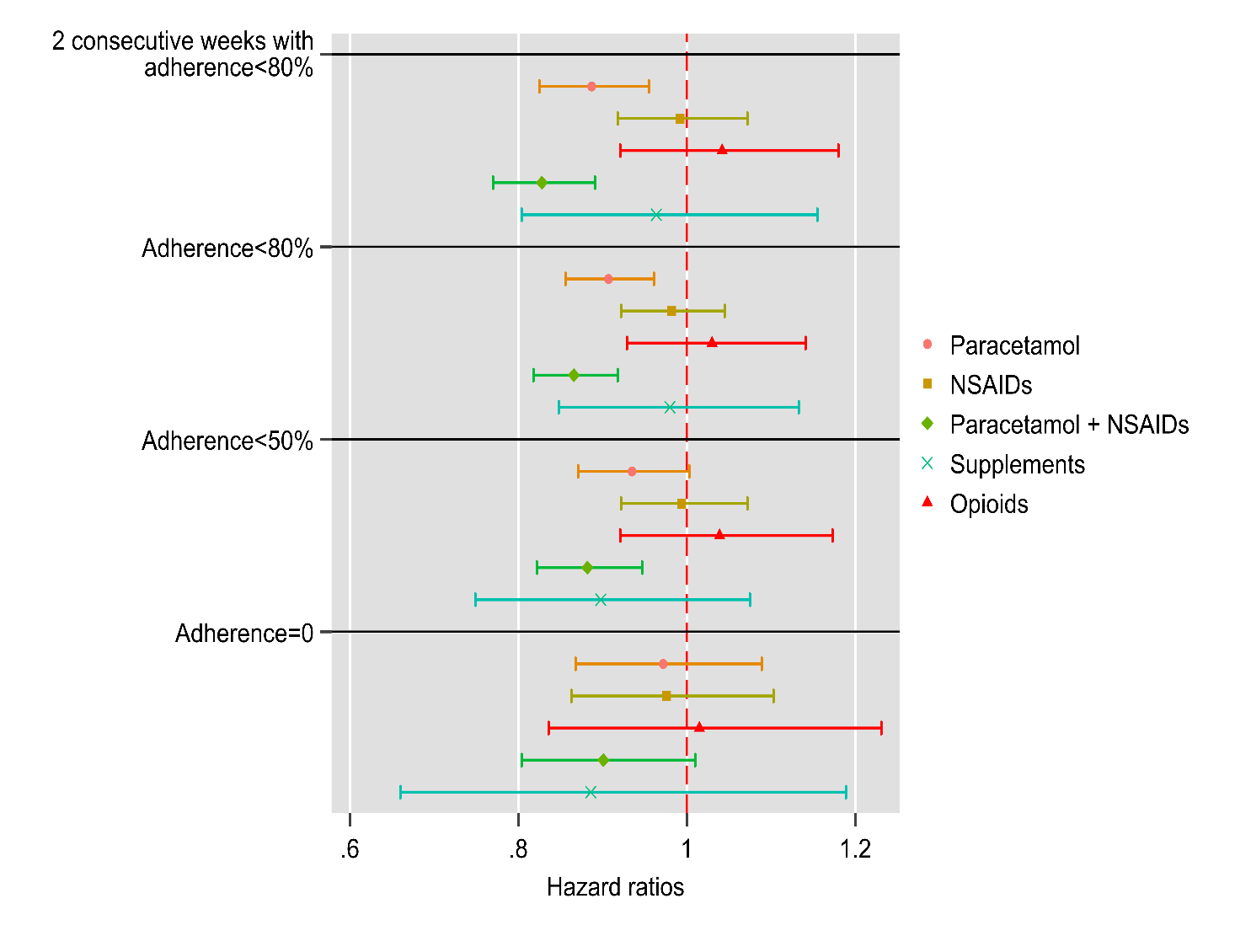


**Table S2. Crude hazard ratios (95% confidence interval) for the associations between medication use and time to reach poor adherence.**

|  | 2 consecutive weeks with adherence<80% | First week with adherence<80% | First week with adherence<50% | First week with adherence of 0% |
| --- | --- | --- | --- | --- |
| No medication (ref) | 1 | 1 | 1 | 1 |
| Paracetamol | 0.918 (0.887, 0.950) | 0.917 (0.889, 0.946) | 0.950 (0.919, 0.981) | 1.001 (0.966, 1.037) |
| NSAIDs | 1.013 (0.974, 1.053) | 0.973 (0.939, 1.008) | 0.988 (0.952, 1.026) | 0.997 (0.957, 1.039) |
| Opioids | 1.260 (1.191, 1.332) | 1.100 (1.044, 1.159) | 1.180 (1.117, 1.246) | 1.208 (1.139, 1.281) |
| Paracetamol + NSAIDs | 0.945 (0.913, 0.978) | 0.922 (0.894, 0.951) | 0.949 (0.918, 0.980) | 1.000 (0.965, 1.036) |
| Supplements | 1.015 (0.925, 1.114) | 1.005 (0.924, 1.094) | 0.957 (0.874, 1.047) | 0.982 (0.890, 1.083) |

**Table S3. Median (95% CI) days to reach poor adherence for different medication categories and differences in median days compared to no medication group, stratified by alternative definitions of adherence.**

|  | 2 consecutive weeks with adherence<80% | | First week with adherence<80% | | First week with adherence<50% | | First week with adherence of 0% | |
| --- | --- | --- | --- | --- | --- | --- | --- | --- |
|  | Median | Difference in median | Median | Difference in median |  |  |  |  |
| No medication (ref) | 44.4 (43.3, 45.4) | 0 | 14.2 (13.7, 14.6) | 0 | 33.8 (32.9, 34.7) | 0 | 71.3 (70.2, 72.4) | 0 |
| Paracetamol | 47.2 (45.8, 48.6) | 2.9 (1.1, 4.7) | 15.7 (15.1, 16.3) | 1.5 (0.8, 2.3) | 36.0 (34.8, 37.2) | 2.2 (0.6, 3.7) | 72.1 (70.8, 73.5) | 0.8 (-0.9, 2.6) |
| NSAIDs | 45.2 (43.5, 46.8) | 0.8 (-1.1, 2.7) | 15.0 (14.3, 15.8) | 0.9 (0.0, 1.7) | 35.3 (33.8, 36.7) | 1.5 (-0.2, 3.2) | 72.4 (70.8, 74.1) | 1.1 (-0.8, 3.1) |
| Opioids | 39.2 (36.9, 41.4) | -5.2 (-7.8, -2.6) | 13.2 (12.2, 14.3) | -0.9 (-2.1, 0.2) | 30.8 (28.7, 32.8) | -3.0 (-5.4, -0.7) | 67.7 (65.2, 70.2) | -3.6 (-6.4, -0.8) |
| Paracetamol + NSAIDs | 49.2 (47.7, 50.7) | 4.9 (3.0, 6.7) | 16.4 (15.7, 17.0) | 2.2 (1.4, 3.0) | 37.6 (36.3, 38.9) | 3.8 (2.2, 5.4) | 73.2 (71.8, 74.6) | 1.9 (0.1, 3.7) |
| Supplements | 46.1 (41.7, 50.6) | 1.8 (-2.8, 6.4) | 14.5 (12.6, 16.3) | 0.3 (-1.6, 2.2) | 37.7 (33.5, 41.9) | 3.9 (-0.4, 8.2) | 74.0 (69.4, 78.6) | - 1. (-2.0, 7.4) |

*all estimates were obtained from parametric interval-censored models adjusted for medication use, age, sex, education, NRS pain, body mass index, sleep rates, EQ-5D-5L index, readiness to do exercise, activity impairment, comorbidity, physical activity, KOOS-12/HOOS-12 total score, walking difficulty, fear of movement, and wish for surgery.
